# Assessment of knowledge, attitude and practice and associated factors of blood donation among health care workers in Ethiopia: a cross-sectional study

**DOI:** 10.1186/s12878-019-0140-9

**Published:** 2019-05-15

**Authors:** Dawit Malako, Fissehatsion Yoseph, Mebratu Legesse Bekele

**Affiliations:** 1 College of Health Sciences and Medicine, Wolaita Sodo University, Wolaita Sodo, Ethiopia; 2 ALERT Hospital, Addis Ababa, Ethiopia

**Keywords:** Knowledge, Attitude, Practice, Health care workers, Blood donation

## Abstract

**Background:**

Blood can only be given from generous donors. The main objective of this study was to assess the knowledge, attitude and practice (KAP) and associated factors of blood donation among health care workers in Wolaita Sodo University Teaching and Referral Hospital (WSUTRH), Wolaita Sodo, Ethiopia.

**Methods:**

An institution-based cross-sectional study was conducted among 218 WSUTRH health care workers. Socio-demographic characteristics and data related to the levels of KAP of participants were collected using a self-administered questionnaire. Bivariate and multivariate logistic regression analyses were conducted using statistical package for social sciences version 20 to assess the factors associated with the practice of blood donation with *p*-value set at < 0.05 for statistical significance.

**Results:**

Two hundred eighteen health care workers were involved in the study among which 129 (59.2%) were males and 89 (40.8%) were females. Among the study participants, 180(82.6%) had good knowledge but only 128(58.7%) were found to have a good attitude as 126(57.8%) reported that voluntary donor is the best source of blood donation. Regrettably, only 47(21.6%) of the respondents were found to practice blood donation in their lifetime. A majority (65.5%) of the participants did not donate blood as they have not been approached to do so. Knowledge and attitude levels of the participants were not found to be significantly associated with sociodemographic parameters study; but, only sex of the participants had shown statistically significant association with blood donation practice where males were more likely to donate blood than females (AOR = 2.59 (1.22–5.49)).

**Conclusions:**

The overall level of knowledge was satisfactory and the level of attitude and practice was unexpectedly low. Female respondents were found to have lesser practice towards blood donation than males. Health care workers, blood banks and the hospital are demanded to design ways to update knowledge, and build its psychological benefits and make services more accessible.

**Electronic supplementary material:**

The online version of this article (10.1186/s12878-019-0140-9) contains supplementary material, which is available to authorized users.

## Background

Blood is a particular body fluid that transports nutrients and oxygen to the cells and moves away the metabolic wastes from the cells. Its transfusion from generous donors is an indispensable part of modern health care which can save lives and improve health. But transfusion of infectious agents carries potential risks up on the receiver demanding particular attention [1–3].

Across the globe, over 80 million units of blood are being donated a year of which only two million in Sub-Saharan Africa where the demand is high [4]. In many developing and transitional countries, there is a wide gap between blood requirements and supplies though its timely accessibility is crucial in all health facilities. (Ambaye Dejen: Knowledge, attitude, practice and factors associated towards blood donation among health care workers in Tikur Anbessa specialized hospital, Addis Ababa, Ethiopia, unpublished) [5]. Developing countries collect less than half of the blood that they need to meet an increasing transfusion requirements due to increasing prevalence of road traffic accidents (RTA), fighting accidents, pregnancy and childbirth, and other medical conditions despite the fact that WHO acclaims its member countries to ensure a safe, adequate and uninterrupted supply of blood and blood products through voluntary unpaid blood donation. In addition, there is often lack of time or proper screening of the donated blood that leaders the general public to emotive and economic stress, significant delays in obtaining safe blood, and risk of blood-borne infections [6–10].

Ethiopian Red-cross society established the national blood transfusion services (NBTS) in 1969 which has been transferred to the Federal Ministry of Health, Ethiopia was entrusted with the responsibility of managing the blood donors, collection, testing and transfusion of blood and blood products in Ethiopia with funding from United States President’s Emergency Plan for AIDS Relief (PEPFAR) through the Centers for Disease Control (CDC) since 2004 [11]. In particular to Ethiopia, some pocket studies found that there was a willingness to donate blood (Daniel Negash: Willingness to voluntarily donate blood among high school students in Addis Ababa: assessment of determinants using the theory of planned behavior, unpublished); but the actual practice of blood donation was not as per the demand where only 43% of blood was being donated though the demand was estimated to be 200, 000 units per annum [12, 13]. As a type of blood being donated, there was a legal framework which precludes the donation other than voluntary basis [14] as per the WHO recommendation being managed by the central Ethiopian national blood bank. Hence, other types of blood donation approaches are banned in Ethiopia and not being used in clinical practice.

Thus, studying knowledge, attitude, and practices (KAP) of health care workers towards blood donation has a great positive impact over the community about the issue either directly or indirectly. They are important in the recruitment of blood donors [15] and their knowledge about blood donation, attitude towards promoting voluntary blood and donation rates (KAP) are important in recruitment of blood donors too [16, 17]. Consequently, they would involve closely and adjust their own behavior and attitude with it as the hospital in which the study was conducted was with a great challenge of blood transfusion service. The possible factors affecting blood donation in the study area would be a benchmark to design evidence-based interventions/education to health care workers that can be delivered and their outcomes (improved health care worker KAP, increased voluntary donations and blood collections). Hence, the main aim of this study was to assess knowledge, attitude and practice and the factors associated with the practice of blood donation among health care workers in Wolaita Sodo University Teaching and Referral Hospital, Wolaita Sodo, Ethiopia.

## Methods

The study was conducted in Wolaita Sodo University Teaching and Referral Hospital, Wolaita Zone, Southern Ethiopia. Wolaita is a zone in Ethiopian Southern Nations, Nationalities, and People’s Region (SNNPR) sharing boundaries in the south with Qucha and Boreda, in the west with Dawro Zone, in the north with Hadya, Kembata and Tembaro Zones and in the east with the Sidama Zone. The administrative center is Wolaita Sodo. It is situated at 380 km to the south of Addis Ababa city, the capital of Ethiopia [18]. The total population is 1.65 million or about 2.31% of the countries’ population; 50.73% being male and 49.27% being female [19]. The Wolaita Sodo University Teaching and Referral Hospital was established in 1928 as a zonal hospital until June 2011 when it was amalgamated into Wolaita Sodo University. The study was conducted from November 1 to 20, 2016. An institution-based cross-sectional study design was used considering the study population being selected health care workers among all health care workers providing health services in Wolaita Sodo University Teaching and Referral Hospital who were available during the data collection period.

To determine the sample size (n) for the study, the single population proportion formula was used. A 5% marginal error (d = 0.05) and by using the maximum proportion of the previous study which was found to have an attitude level of 68% (Ambaye Dejen: Knowledge, attitude, practice and factors associated towards blood donation among health care workers in Tikur Anbessa specialized hospital, Addis Ababa, Ethiopia, unpublished) to increase the power of the study with assumption of confidence interval to be 95% (Z^2^α/2 = 1.96) was used to determine the minimum possible sample size. Using the formula for finite population (i.e. a total of 500 health care workers in the hospital (N)), the sample size was calculated as depicted hereunder.

$$ \mathrm{n}=\left({{\mathrm{Z}}^2}_{\upalpha /2}\ \mathrm{p}\ \left(1-\mathrm{p}\right)\ \mathrm{N}\right)/\left({\mathrm{d}}^2\mathrm{N}+{{\mathrm{Z}}^2}_{\upalpha /2}\ \mathrm{p}\ \left(1-\mathrm{p}\right)\right) $$

$$ \mathrm{n}=1.96\ast 1.96\ast .68\ast \left(1-.68\right)\ast 500/\left(0.05\ast 0.05\ast 500+1.96\ast 1.96\ast .68\ast \left(1-.68\right)\right)=201 $$

Considering a 10% non-response rate, the total sample size was corrected to be 201+ (201*0.1) = 222.

**Fig. 1:**
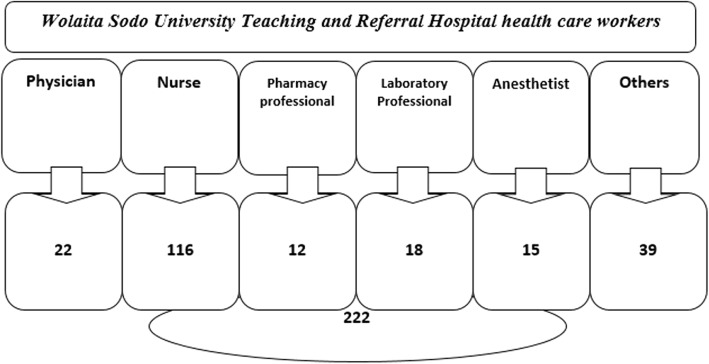
Schematic illustration of samples selected

With regard to the sampling procedure, the study subjects were selected using stratified sampling method in such a way that sample was stratified based on their departments proportionally depending upon the number of health care workers in each department with a rationale to assess any possible association of KAP of blood donation with the health care workers of different departments and then from each strata (departments) the proportionally allotted number of respondents were drawn by using simple random sampling (lottery) technique. Accordingly, the calculated 222 study participants were selected from each department as depicted in Fig. 1.

Data were collected by using a self-administered questionnaire which was prepared in English. The questionnaire was made include sociodemographic characteristics of the participants like age, sex, religion, marital status, department, qualification, monthly income, work experience, mass media exposure and questions/parameters which were designed to assess the knowledge, attitude, and practices of blood donation.

To assure the quality of data, a structured questionnaire which was carefully adopted without changing the original meaning and context from published journals was used for this study [4, 10, 20]. On its top, the questionnaire was pretested in Dubbo St. Mary Hospital health care workers and was checked for its clarity, understandability, and completeness based on objectives and variables before distributing to the actual respondents.

After the questions and the variables were checked and ascertained for completeness, consistency, and accuracy, the data were cleaned, coded, entered, and analyzed by using statistical package for social sciences (SPSS) version 20 software. Descriptive statistics like percentage, ratio, mean, median and frequency were done. Odds ratio with 95% CI was used to measure the association of socio-demographic and other independent variable related to the practice of blood donation. Multivariable logistic regression analysis was used to assess an independent association of predictor variables with the outcome variable.

### Operational definitions

#### Health care worker

Is health professional that provides preventive, curative, promotional or rehabilitative health care services for individuals or community.

#### Level of attitude

Attitude is the intention of respondents of the study towards the blood donation practice. The attitude of blood donation was assessed through six questions. Those who scored less than the 50th percentile were categorized as having a poor attitude whereas those who scored 50th percentile or more were categorized as having a good attitude towards blood donation.

#### Level of knowledge

This is the understanding level of health care workers on blood donation. Based on the score of response knowledge level was categorized into having a good knowledge or poor knowledge. Knowledge level of the study subjects was assessed by using ten questions. Those who scored less than the 50th percentile were categorized as having a poor knowledge and those who scored the 50th percentile or more were categorized as having a good knowledge.

#### Paid or remunerated donors

Paid donors are individuals who give blood in return for money or other forms of payment.

#### Practice of blood donation

Practice towards blood donation denotes an experience whether an individual participant had experienced blood donation at least once in a lifetime or not at all. Those who donate blood at least once in a lifetime were categorized as having good practice whereas those who never donated blood at all in a lifetime were categorized as having poor practice towards blood donation.

#### Regular donor

Regular blood donor is an individual who donates blood voluntarily every 3–4 months.

#### Replacement donor

Replacement donor is a donor of blood for relatives or friends to replace blood used from blood bank stocks.

## Results

Out of the calculated sample size of 222, 218 were involved in the study with a non-response rate of 1.8%. Among them, 129(59.2%) were males and 89(40.8%) were females. Nearly half of the respondents (46.8%) were in the age group of 26–30 years and the minimum and maximum were 20 and 50 years respectively. Approximately half (107 (49.1%)) were single and 106 (48.6%) were married. Protestant Christianity and Orthodox Christianity were the predominant religions of the study subjects being represented by 107 (49.1%) and 89 (40.8%) respectively. Ninety-seven (44.5%) served 1–2 years in the health facility where they were working during the study period and most of the participants were nurses (Table 1).

**Table 1:** Socio-demographic characteristics of WSUTRH health care workers towards blood donation, 2017

Variable	Category	*N*	%
Age	20–25	61	28
	26–30	102	46.8
	31–35	33	15.1
	36–40	15	6.9
	> 41	7	3.2
Sex	Male	129	59.2
	Female	89	40.8
Marital status	Single	107	49.1
	Married	106	48.6
	Divorced	4	1.8
	Widowed	1	0.5
Qualification	Diploma	77	35.3
	First degree	138	63.3
	Second degree	1	0.5
	Specialist	2	0.9
Religion	Protestant	107	49.1
	Orthodox	89	40.8
	Catholic	9	4.1
	Muslim	6	2.8
	Other	7	3.2
Duration of practice in years	1–2	97	44.5
	3–4	31	14.2
	5–6	23	10.6
	7–8	31	14.2
	9–10	16	7.4
	≥11	20	9.1
Department	Nursing	116	53.2
	Physician	22	10.1
	Laboratory	18	8.3
	Anesthetists	15	6.9
	Pharmacy	8	3.7
	Others	39	17.9
Monthly income in birr	1000–3000	87	39.9
	3001–6000	81	37.2
	6001–9000	48	22
	> 9000	2	0.9
Easy accessibility of blood bank	Yes	26	11.9
	No	192	88.1

More than three-fourths of respondents, 169 (77.5%) itemized that HIV, HBV, and HCV can be transmitted through blood donation whereas 17 (7.8%) of the respondents listed that syphilis, malaria, and CMV can be transmitted through blood transfusion. One hundred forty (64.2%) did know how often an individual donates blood and 78 (35.8%) did not know the interval of blood donation. Twenty-three (10.6%), 54 (24.8%) and 146 (66.1%) did not know who should donate blood, the volume of blood collected during each donation and the duration of donation process respectively. In general, 180(82.6%) of respondents were found to have good knowledge of blood donation (Table 2).

**Table 2:** Level of knowledge of WSUTRH health care workers towards blood donation, 2017

Questions	Category	*N*	%
Do you know common blood groups?	Yes	215	98.6
	No	3	1.4
Do you know your blood group?	Yes	205	94
	No	13	6
What is your blood group?^b^	A+	40	19.5
	A-	10	4.9
	B+	50	24.4
	B-	10	4.9
	AB+	19	9.2
	AB-	8	3.9
	O+	59	28.8
	O-	9	4.4
Can a person be infected by receiving blood transfusion?	Yes	190	87.2
	No	28	12.8
What diseases are transmissible by blood transfusion?	HIV, HBV &HCV	169	77.5
	Syphilis, Malaria, and CMV	17	7.8
	Other diseases^a^	32	14.7
How often an individual donate blood?	Monthly	3	1.4
	Three monthly	140	64.2
	Six monthly	58	26.6
	Annually	12	5.5
	I don’t know	5	2.3
Who should donate blood?	Men, women & healthy	195	89.4
	Old > 60 yrs., young< 18 yrs. vulnerable group & diseased	23	10.6
What volume of blood is collected during each donation?	< 500 ml	164	75.2
	500-1000mls	45	20.7
	I Don’t know	9	4.1
What is the duration of the donation process?	20 min	74	34
	20–60 min	118	54.1
	I don’t know	26	11.9
What is your primary source of information about blood donation?	Training/school/college	112	51.4
	Health facility	53	24.3
	Mass media	39	17.9
	Others	13	6
	Have no information	1	0.5

^a^Typhoid fever, Typhus fever, Relapsing fever ^b^Thirteen respondents did not know their blood group

Among the study participants, 128(58.7%) were found to have a good attitude towards blood donation. Almost all of the respondents, 216 (99.1%), responded that blood donation is good. But only 126(57.8%) reported that voluntary donor is the best source of blood donation and around one-third, 71 (32.6%) responded that it is a replacement donor. Temporary weakness was commonly assumed to happen to a donor after donation (175(80.3%)). One hundred sixty-six (76.1%) reported that the relative of the patient should be asked to donate (Table 3).

**Table 3:** Level of attitude of WSUTRH health care workers towards blood donation, 2017

Questions	Category	*N*	%
What do you think about blood donation?	Good	216	99.1
	Bad	–	–
	No idea	2	0.9
What do you think is the best source of blood donation?	Voluntary	126	57.8
	Replacement	71	32.6
	Remunerated	2	0.9
	Self-donor	17	7.8
	I don’t know	2	0.9
Can something harmful happen to a blood donor during or after donation?	Yes	149	68.3
	No	57	26.1
	I don’t know	12	5.5
What can happen to a blood donor during or after donation?	Contract infection	24	11.6
	Temporary weakness	175	80.3
	Fall sick	7	3.4
Should patient relative be asked to donate?^a^	Yes	166	76.1
	No	50	22.9
	I don’t know	2	0.9
Do you encourage relatives to donate?	Yes	136	62.4
	No	82	37.6
Will you donate if called upon or reminded to do so?	Yes	123	56.4
	No	95	43.6

^a^Twelve respondents didn’t answer

With regard to blood donation practice, only 47 (21.6%) were found to donate blood and only 6 (2.8%) donated more than three times in their lifetime. Among the study participants who had blood donation practice, more than half of the participants practiced blood donation were in age groups less than 30 years and only 27 (12.4%) were of their own free will. Out of the respondents those who did not donate blood, most (112 (65.5%)) reasoned as they were not approached to donate. In addition, fear of needle and fear of knowing their own sero-status were among the reasons described by the participants that could hinder donation. Among all non-donors, 25 (11.5%) responded that they need to donate to friends or relatives in the future (Table 4).

**Table 4:** Level of practice of WSUTRH health care workers towards blood donation, 2017

Questions	Category	*N*	%
Have you ever donated blood?	Yes	47	21.6
	No	171	78.4
How often do you donate in a year?	Once	28	12.8
	2–3 times	13	6
	More than 3 times	6	2.8
Why did you donate?	A friend or relative needed blood	20	9.2
	Voluntarily	27	12.4
	Remunerated (paid)	–	–
	To know my screen status	–	–
Reasons for non-donation by non-donors	Not approached to donate	112	51.4
	Unfit to donate	7	3.2
	Fear of needle	15	6.9
	Fear of knowing my status	10	4.6
	Religion forbids it	1	0.5
	Donated blood may be sold	–	–
	No remuneration (payment)	1	0.5
	Need to donate to friends or relatives in the future	25	11.5

Knowledge and attitude levels of the respondents were not found to have statistically significant association with sociodemographic parameters study; but, only sex of the respondents had shown statistically significant association with the practice of blood donation among Wolaita Sodo University Teaching and Referral Hospital health care workers that males were more likely to donate blood than females [AOR (95% CI): 2.59(1.22–5.49)].

## Discussion

In this institution based cross-sectional study on KAP and associated factors towards blood donation of WSUTRH health care workers, a total of 218 study subjects were involved. Among the respondents, 102 (46.8%) were in the age group of 26–30 years. The mean and the median age of the respondents were 28.8 and 28 years respectively with a standard deviation of 5.22. This socio-demographic fact of the study result shown that the population was built with younger workers in contrary to some developed countries. This was a very great opportunity to expand blood donation services. It was relatively comparable with the result of the study conducted in Kenya in which most of the blood donors in Nairobi found to be in age between 18 and 28 years [21]. This is an opportunity for blood donation in our study area as opposed to the studies conducted in Australia, Canada and America aging being among the major challenges of blood donation [1, 22, 23].

As of the finding of this study, the level of knowledge on blood groups and how often an individual can donate blood was good in comparison to the result of the study conducted in Addis Ababa health facilities with this regard [8]. In our study, most of the health care workers had knowledge that a person can be infected by receiving a blood transfusion. On the contrary, in a study conducted in the democratic republic of Congo on assessment of KAP regarding blood donation, 61% did not know the practice of donating blood and the knowledge of blood donation and it was significantly associated with educational level of the respondents and their religion [24] which was not in our study case. Besides this, our study result was much higher than the report of 2015 WHO regional office for Africa average prevalence of adequate knowledge towards blood donation and the study conducted in Samara, Ethiopia [25, 26]. Unlike the study conducted in Addis Ababa, in this cross-sectional study, there was no significant statistical association of the level of knowledge with the sex of the respondent and other variables (Ambaye Dejen: Knowledge, attitude, practice and factors associated towards blood donation among health care workers in Tikur Anbessa specialized hospital, Addis Ababa, Ethiopia, unpublished).

On the assessment of attitude level, 58.5% of the respondents were found to be with a good attitude towards blood donation. This result was lower than the result of the studies conducted in Addis Ababa (Ambaye Dejen: Knowledge, attitude, practice and factors associated towards blood donation among health care workers in Tikur Anbessa specialized hospital, Addis Ababa, Ethiopia, unpublished) [8]. Even though 99.1% responded that blood donation is good, only 57.8% reported that voluntary donor is the best source of blood donation as of our study finding. This was much below the WHO recommendation of 100% unpaid voluntary blood donation [23] though higher than the result of a study conducted in Nigeria [4]. Unexpectedly, nearly one third (32.6%) of the respondents answered the replacement donor as if the best source of blood which ratified the declined attitude of the respondents as they were health care professionals. On its top, the study participants’ willingness to donate blood was found to be low as compared to the result of the study conducted at Ambo town in which 100% of the respondents were willing to donate blood in the future and 70% in the study conducted in Addis Ababa [11] (Daniel Negash: Willingness to voluntarily donate blood among high school students in Addis Ababa: assessment of determinants using the theory of planned behavior, unpublished).

In this study, only 21.6% of participants were found to have good practice towards blood donation and age groups less than 30 years and physicians were more frequently donating than others. The practice of blood donation was too much lower when compared to the practices in Austria (66%), France (52%), Greece and Cyprus (51%) [22, 27]. It was nearly similar to the result of the studies conducted in Ambo University, Gondar and Addis Ababa, Ethiopia (Ambaye Dejen: Knowledge, attitude, practice and factors associated towards blood donation among health care workers in Tikur Anbessa specialized hospital, Addis Ababa, Ethiopia, unpublished) [11, 16]. These discrepancies and similarities might be due to the socioeconomic status and awareness level of the participants as the practice of blood donation was depicted to be higher in developed countries aforementioned.

Religious background of the health care workers did not affect their attitude towards or likelihood of donating blood in our study; rather, sex of the respondents had a statistically significant association with the practice of blood donation where males were more likely to donate blood than females [AOR (95% CI): 2.59(1.22–5.49)]. This finding was in concordance with the findings of a study conducted in Nigeria [28]. Perhaps, this might be due to the burden of females as a matter of their extensive involvement in routine household activities with low nutritional security in the study area and lower hemoglobin levels due to menses paving a way for the development of anemia precluding blood donation [29]. Added to this, among the respondents never experienced blood donation in their lifetime, 65.5% didn’t practice blood donation because they were not approached to donate. This was relatively congruent to the result of the study conducted in Cameroon in which 60% had not previously been asked to donate blood [30].

Therefore, pieces of trainings have to be facilitated by the institution for health care workers to update their knowledge and attitude with the increasing demand for blood transfusion with due motivational schemes. On its top, the blood banks should facilitate pieces of trainings and make the services more accessible to health care workers in order to get regular blood donors.

However, the present study has some expected limitations. The limitation of the study could be the cross-sectional design used which might not define the cause-effect relationship of the factors with the KAP of blood donation, the use of only quantitative data without triangulating with qualitative due to resource shortage, and not using a validated questionnaire for data collection.

## Conclusions

According to this institution-based cross-sectional study, even though the overall level of knowledge was satisfactory, nearly half of the respondents’ attitude on blood donation was unexpectedly poor. The willingness to donate blood if called upon or reminded to do so and the practice of blood donation were also poor. Female respondents were found to have poor practice towards blood donation than males. Not approaching to donate was the leading reason for non-donors. Hence, health care workers are demanded to update their knowledge and build psychological benefits of blood donation in order to save lives. On its top, blood banks have to make their services more accessible to health care workers in order to get regular blood donors. Further studies should be conducted on the KAP of blood donation from the general public angle so that the service be scaled up as per the increasing demand.

## Additional file

Additional file 1: Questionnaire. The self-administered questionnaire used for assessment of knowledge, attitude and practice and associated factors of blood donation among health care workers in Ethiopia. (PDF 305 kb)

## Abbreviations

AIDS: Acquired immune deficiency syndrome
CDC: Centers for disease control
CMV: Cytomegalovirus
HCV: Hepatitis C virus
HIV: Human immune-deficiency virus
KAP: Knowledge, Attitude, and practice
NBTS: National blood transfusion services
PEPFAR: President’s emergency plan for AIDS Relief
RTA: Road traffic accidents
SNNPR: Southern nations, nationalities people region
WHO: World Health Organization
WSUTRH: Wolaita Sodo University Teaching and Referral Hospital
