# Supplementary material for: Assessment of knowledge, attitude and practice and associated factors of blood donation among health care workers in Ethiopia: a cross-sectional study
Source: BMC Hematol. 2019 May 15;19:10. doi: 10.1186/s12878-019-0140-9 (PMC6521463; doi:10.1186/s12878-019-0140-9)
Supplement: Supplementary file 1 — Questionnaire. The self-administered questionnaire used for assessment of knowledge, attitude and practice and associated factors of blood donation among health care workers in Ethiopia. (PDF 305 kb) [file 12878_2019_140_MOESM1_ESM.pdf]

Questionnaire

We are working on a research entitled knowledge, attitude, and practice and associated factors towards blood donation.

You have been selected to participate in this study. The finding of the study will be used for better planning and up on intervention towards blood donation concerning the awareness, perception and practice. Therefore we are requesting you to fill this questionnaire by yourself. There is no right or wrong answer. Your response is completely confidential. You do not need to write your name and will never be used in a connection with any of the information you will give. You are kindly requested to answer every question and may stop filling at any time if you want to do so. However, your honest answers to these questions will help us better understand what people think, say and do about the blood donation. According to the questions, there might have more than one answers.

WE would greatly appreciate your help in responding to these questions. The survey will take about 30 minutes to fill this questionnaire.

Are you willing to participate? Yes/ No

Please read each questions carefully and show your answer by circling the number and writing your response on blank spaces.

Thank you in advance for your participation!

Part I: Socio-demographic questions

101	Age	………….years
102	Sex	Male Female
103	Marital status	Single Divorced<br>Married Widowed
104	Qualification	Diploma 2nd degree<br>First degree Specialist
105	Religion	Orthodox<br>Muslim<br>Catholic<br>Protestant<br>Others…………….
106	Duration of practice in the health facility	……………Years/…………Months
107	Department	Physician<br>Nursing<br>Laboratory<br>Pharmacy<br>Physiotherapy<br>Anesthesia
108	Monthly income	………………..birr
109	Is blood bank easily accessible around your institution?	Yes<br>No

Part II: knowledge on blood donation

201	Do you know common blood groups?	Yes<br>No
202	Do you know your blood group?	Yes<br>No
203	What is your blood group?	A+<br>A-<br>B +<br>B -<br>AB+<br>AB-<br>O+<br>O-
204	Can a person be infected by receiving blood transfusion?	Yes<br>No
205	What diseases are transmissible by blood transfusion?(Please list)	
206	How often an individual donate blood?	Weekly<br>Monthly<br>Three monthly<br>Six monthly<br>Annually<br>Don’t know
207	Who should donate blood? (Please list)	

208	What volume of blood is collected during each donation?	<500ml<br>500-1000ml<br>Don’t know
209	What is the duration of a donation process?	20 min<br>20-60min<br>don’t know
210	What is your source of information about blood donation?	Health facility<br>Family<br>Friends<br>Mass media<br>Training/School/College<br>Others…………..<br>Have no information

PART III: attitude on blood donation

311	What do you think about blood donation?	Good<br>Bad<br>No idea
312	What do you think is the best source of blood donation?	Voluntary donor<br>replacement donor<br>remunerated donor<br>self-donor<br>don’t know
313	Can something harmful happen to a blood donor during or after blood donation?	Yes<br>No<br>I don’t know
314	What can happen to a blood donor during or after donation?	Contract infection<br>temporary weakness<br>fall sick

315	Should patient relative be asked to donate?	Yes<br>No<br>I don’t know
316	Do you encourage relatives to donate?	Yes<br>No

PART IV: Practice of blood donation

401	Have you ever donated blood in your life time?	Yes<br>No
402	How often do you donate in a year?	……………… times in a year.
403	Why did you donate?	A friend or relative needed blood<br>Voluntarily<br>Remunerated(paid)<br>To know my screen status
404	Reasons for not donating by nondonors?	Not approached to donate<br>Unfit to donate<br>Fear of needle<br>Fear of knowing my status<br>Religion forbid it<br>Donated blood may be sold<br>No remunerations(payment)<br>Need to donate to friends or relative in the future

THANK YOU!!
